# Elevated Tumor Cell-Intrinsic STING Expression in Advanced Laryngeal Cancer

**DOI:** 10.3390/cancers15133510

**Published:** 2023-07-05

**Authors:** Jelena Viculin, Marina Degoricija, Katarina Vilović, Ivana Gabela, Lucija Franković, Eduard Vrdoljak, Jelena Korac-Prlic

**Affiliations:** 1 Department of Oncology and Radiotherapy, University Hospital of Split, 21000 Split, Croatia; jviculin@kbsplit.hr (J.V.);; 2 Department of Medical Chemistry and Biochemistry, School of Medicine, University of Split, 21000 Split, Croatia; 3 Department of Pathology, Forensic Medicine and Cytology, University Hospital of Split, 21000 Split, Croatia; 4 Department of Anatomy, School of Medicine, University of Split, 21000 Split, Croatia; 5 Laboratory for Cancer Research, Department of Immunology and Medical Genetics, School of Medicine, University of Split, 21000 Split, Croatia; 6 Department of Clinical Oncology, School of Medicine, University of Split, 21000 Split, Croatia

**Keywords:** laryngeal cancer, cGAS–STING pathway, STING agonist, PARP inhibitors, chemoradiotherapy

## Abstract

**Simple Summary:**

Novel therapeutic approaches are required to improve the outcomes of immunotherapy for laryngeal cancer. The immunomodulatory effects of the DNA sensor cGAS and the cyclic GMP–AMP receptor stimulator of interferon genes (STING) signaling axis have been extensively studied in various types of cancer; however, their role in laryngeal cancer remains unknown. The findings of this study demonstrated that STING is upregulated in immunologically active advanced laryngeal cancer. Targeting the STING–cGAS signaling pathway in laryngeal cancer might potentially improve current therapeutic approaches, and elevated STING expression could be considered as a predictive biomarker in future clinical trials, including STING agonists.

**Abstract:**

Laryngeal cancer is the second most common malignancy of the head and neck, worldwide. Immunotherapy targeting checkpoint inhibitors has been approved for the treatment of patients with recurrent or metastatic laryngeal cancer but has a relatively low response rate and outcomes that leave many patients underserved. Targeting the cGAS–STING signaling pathway can potentially improve the activation of immune effector cells, although its role in the development and progression of laryngeal cancer has not yet been investigated in depth. Fifty-nine tumor samples from patients with pathologically confirmed squamous cell carcinoma of the larynx, stage I–IV non-metastatic disease, who were treated at the University Hospital of Split, were immunohistochemically stained for the expression of STING, cGAS, CD8, CD68, and CD163. Elevated tumor cell-intrinsic STING expression was positively associated with stage IV (*p* = 0.0031), pT3, and pT4 laryngeal cancers (*p* = 0.0336) as well as with higher histological grades (G2 and G3) (*p* = 0.0204) and lymph node-positive tumors (*p* = 0.0371). After adjusting for age, sex, location, and cGAS expression, elevated STING expression was significantly associated with stage IV cancer in a multiple logistic regression model (β = 1.849, SE = ±0.8643, *p* = 0.0324). Elevated STING expression represents a potentially favorable predictive biomarker for new therapeutic approaches involving STING agonists combined with immunotherapy and DNA-damaging agents (radiotherapy, cisplatin, and PARP inhibitors) in laryngeal cancer.

## 1. Introduction

Head and neck tumors account for approximately 3–5% of all malignant diseases, and laryngeal cancer is the second most common, accounting for approximately 30% of all newly diagnosed head and neck tumors. Although the incidence of laryngeal cancer is declining worldwide, according to the International Agency for Research on Cancer, it is estimated that more than 184,000 people were diagnosed with laryngeal cancer in 2020 and almost 100,000 people died from this disease worldwide. More than 90% of all malignant tumors of the head and neck are squamous cell carcinomas [1]. The five-year relative survival rate is approximately 79% for localized disease, less than 50% for regionally advanced disease, and 35% for metastatic disease [2]. Depending on the disease stage, tumor location, and general health status of the patient, the available therapeutic options include surgical treatment, radiation, chemotherapy, biological therapy, immunotherapy, or a combination of these, always considering the importance of organ preservation. In the early stages of the disease, single-modality treatment, radiation, or surgery is equally effective for local disease control and overall survival (OS). For radiation therapy, the ten-year disease-specific overall survival rates are >90% and 70% for stages one and two, respectively [3]. Approximately 30–40% of laryngeal carcinomas are diagnosed at a locoregionally advanced stage [2]. In these patients, preference is given to therapeutic options that preserve the glottis if possible. The treatment for locoregionally advanced stages of the disease includes combination treatment, chemoradiotherapy, and surgery with radiation. Based on the positive results of clinical studies, immunotherapy with PD1 and PD-L1 checkpoint inhibitors, alone or in combination with chemotherapy, has been approved for the treatment of patients with recurrent or metastatic laryngeal cancer since 2016 [4]. The use of immunotherapy significantly prolongs the median overall patient survival, and an excellent response with long–term disease control has been achieved in a small number of patients [5]. However, there is still a great need for improvements in the therapeutic outcomes of laryngeal cancer patients regarding organ preservation, as well as improvements in overall survival for patients with metastatic stages of the disease [5,6]. Unfortunately, an epidemiological study in the United States showed a decrease in 5-year survival from 66 to 63% in the last 40 years, most likely due to more frequent organ preservation in the treatment of advanced laryngeal cancer, defining a need for better, optimized, and multidisciplinary treatment approaches with potentially better outcomes as a final result [7].

The inclusion of immunotherapy in standard treatment protocols for advanced laryngeal cancer suggests its possible application in the early stages of the disease, aiming for better systemic and local disease control [8]. This concept has proven successful in the neoadjuvant treatment of a series of patients with solid tumors deficient in mismatch repair (dMMR) [9,10,11]. Malignant head and neck tumors show abundant infiltration of immune cells as well as high mutational and antigen load [12]. However, the response to immunotherapy in advanced and metastatic disease is modest; therefore, a better understanding of the immune process and precise predictive biomarkers are required for successful patient selection. Several ongoing clinical and recently published studies have investigated the use of immunotherapy alone or in combination therapy in the early stages of the disease [8,13].

Stimulator of interferon genes (STING) is a protein that is expressed mostly in the endoplasmic reticulum of various cell types. It is a component of the cGAS–STING signaling cascade responsible for the stimulation of the innate immune response to foreign DNA in the context of infection, cellular stress, and tissue damage [14]. cGAS is a direct cytosolic double-stranded DNA (dsDNA) sensor crucial for activating the type I interferon response to invading DNA viruses or bacteria; however, it also plays an important role in cancer development [15]. Activation of the cGAS–STING pathway in cancer influences the polarization of pro-tumorigenic macrophages M2 into anti-tumorigenic macrophages M1, attracts CD8+ lymphocytes, and reduces the expression of PD-L1 in tumor tissues [16]. The anticancer immunity exerted by the cGAS–STING pathway relies on the successful activation of immune effector cells. However, it can also induce an immunosuppressive response through immune checkpoints (such as PD-L1), depending on the context of the tumor microenvironment [17]. Impaired cGAS–STING signaling is associated with poor prognosis in multiple solid tumor types as well as low immune cell infiltration [18,19,20,21,22]. Although head and neck cancers generally have abundant immune cell infiltration and a high mutational burden, the response to immunotherapy alone or in combination with chemotherapy is low, with only approximately 15–20% of patients with metastatic disease [8]. A recent cGAS–STING prediction model defined biomarker genes that stratify tumors, including bladder cancer, lung adenocarcinoma, lung squamous cell carcinoma, skin cutaneous melanoma, and ovarian cancer, from the TCGA database into four distinct cGAS–STING groups (CSG 1–4) according to the clinical significance of cGAS–STING signaling in immunotherapy response. Patients with high cGAS–STING functional activation showed an improved response and prognosis in the bladder cancer immunotherapy cohort, which was related to TP53 mutational status, chromosomal instability, and tumor neoantigen burden [23]. Modulation of the cGAS–STING signaling pathway has potential therapeutic benefits in different types of malignant tumors, and it is currently being investigated in numerous clinical studies, but its role in the development and progression of laryngeal cancer has not yet been investigated [13]. Therefore, this study aimed to assess the expression levels of cGAS and STING in laryngeal cancer in both early and advanced non-metastatic stages of the disease.

## 2. Methods

### 2.1. Patients

This single-center retrospective study included all patients with pathohistologically confirmed squamous cell carcinoma of the larynx, stage I–IV non-metastatic disease at the time of diagnosis, who underwent surgery at the University Hospital of Split in Croatia and were referred to the Department of Oncology and Radiotherapy between January 2015 and December 2018. A total of 59 patients were identified by searching the archived medical records of the Department of Oncology and Radiotherapy. Data relating to demographic, clinical, and pathological features as well as outcomes were collected and are presented in Table 1. Pathological diagnosis was independently confirmed by two experienced pathologists. The clinical follow-up of the patients was conducted on 31 October 2022.

Exclusion criteria: Patients with cancer of the larynx, which is not of squamous differentiation, or with initial stage IV metastatic disease, those who were diagnosed or treated outside the University Hospital Split, and those with carcinomas of squamous differentiation located outside the larynx. In addition, patients who were not candidates for treatment other than palliative care due to age and other comorbidities or who refused the proposed treatment were excluded from the study. All treatment decisions were made by a multidisciplinary team according to relevant guidelines.

The Ethics Committees of the University Hospital of Split and the School of Medicine in Split have approved this cohort study (Klasa:500-03/22-01/72, Ur.br.:2181-147/01/06/M. S.-22-02, 9 May 2022).

### 2.2. Immunohistochemistry

Immunohistochemical staining of 4 µm-thick formalin-fixed, paraffin-embedded tissue sections was performed as follows. Slides were dried at 60 °C, deparaffinized in xylene, and rehydrated using graded alcohol solutions in water. Heat-induced epitope retrieval was performed by boiling the sections in EDTA buffer (pH 9.0) in a microwave. The sections were left at room temperature for 20 min, rinsed with water, and placed in Tris-buffered saline (TBS) for 5 min. Peroxidase blocking solution (EnVision kit, Dako-Cytomation, Glostrup, Denmark) was used for 15 min to block endogenous peroxidase activity. After the tissue sections were rinsed with TBS, the slides were incubated for 1 h and 30 min with either a primary polyclonal mouse anti-human c-GAS antibody (Proteintech, Rosemont, IL, USA, 26416-1-AP, dilution 1:200) or a STING antibody (Proteintech, 19851-1-AP, dilution 1:2000). Tissue sections were incubated with secondary antibodies (EnVision, Shanghai, China) and reviewed using the OptiView DAB IHC v6 kit (Roche Diagnostics, Switzerland). The sections were stained with hematoxylin, dehydrated, and mounted. The immunostaining using CD8 antibody (Ventana, Cupertino, CA, USA, SP57, 790-4460, dilution 1:50), CD68 antibody (Ventana, PG-M1, M0876, dilution 1:50) and CD163 antibody (Ventana, MRQ-26, 163M-15, dilution 1:50) was performed on Ultra Benchmark (Ventana Medical Systems, Inc., Oro Valley, AZ, USA), according to the manufacturer’s instructions.

### 2.3. Immunohistochemical Staining

**Figure 1:**
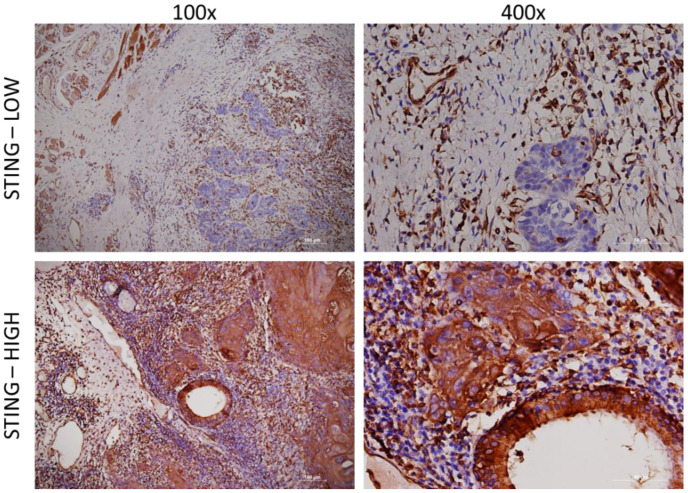
STING immunostaining. Representative histologic images of low and high expression of STING at 100× (**left**) and 400× (**right**) magnification.

**Figure 2:**
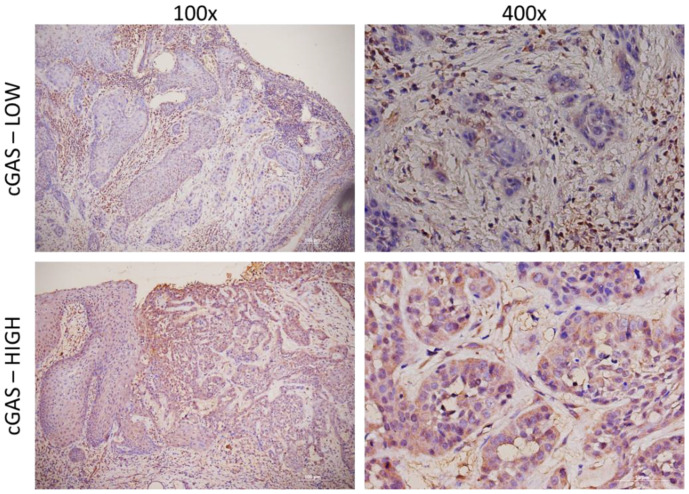
cGAS immunostaining. Representative histologic images of low and high expression of cGAS at 100× (**left**) and 400× (**right**) magnification.

Two independent experts, pathologists, and experienced scientists semi-quantitatively assessed the IHC expression levels of cGAS and STING proteins by integrating the percentage and intensity of immunostaining of cancer cells. Immunoreactivity intensity was adjusted to the baseline staining of the epithelium and glandular tissue and was marked as high or low (Figure 1 and Figure 2) [19,24]. All samples were analyzed using hematoxylin and eosin staining and immunohistochemical staining for CD8, CD68, and CD163 to determine the relative abundance of immune cells in the tumor stroma.

To evaluate CD8+, CD86+, and CD163+ immune cells, the number of positive cells was calculated using IHC staining. Each sample was screened at a low magnification (40×), and the area with the greatest number of positively stained cells in the stroma was selected for further analysis. Each sample contained a minimum of 50 CD8+, CD68+, or CD163+ positively stained cells. Semi-quantitative analysis (0, +, ++, +++) of the hotspots was performed at high magnification (400×).

### 2.4. Statistics

Categorical variables were presented as numbers and percentages, whereas patient age and overall survival (OS) were presented as medians with interquartile ranges (IQR). Fisher’s exact test and chi-square (χ^2^) test were used to test the independence of two or more categorical variables, respectively. Associations between categorical variables were calculated using Spearman’s correlation coefficient. Additionally, multiple logistic regression was used to model the probability of increased STING expression in advanced stages of laryngeal cancer. Statistical significance was defined as *p* < 0.05. Data were analyzed using GraphPad Prism (version 9.5.1., La Jolla, CA, USA).

## 3. Results

Tissue samples from 59 patients with laryngeal SCC were included in this study, with a median follow-up period of 64 (49–73) months. The clinicopathological features of the patients are presented in Table 1. The median age of the patients upon diagnosis was 65 years, and the majority of patients were male (94.9%). There were 11 (18.6%) supraglottic tumors, 46 (78.0%) glottic tumors, and 2 (3.4%) subglottic tumors, of which 22 (37.3%) were transglottic tumors, mainly derived from the glottis as the primary site (20 22). Sixteen tumors (27.1%) were well-differentiated, 37 (62.7%) were moderately differentiated, and six (10.2%) were poorly differentiated. Fifteen (25.4%) tumor cases had lymphovascular invasion and seven (11.9%) had perineural invasion. Sixteen (27.1%) patients had lymph node metastases in a surgical specimen and five (8.5%) patients had N1 disease. Advanced disease stage (stages III and IV) was observed in 36 patients (61.1%). Three patients received preoperative radiotherapy, 33 patients who received adjuvant therapy were treated with radiotherapy only, and four patients received cisplatin-based concurrent chemoradiotherapy. Local recurrence of the disease was confirmed in four (6.8%) patients and distant metastases in four (6.8%) patients. Another primary malignant tumor was detected in 14 patients (23.7%). The median overall survival was not reached, and the median disease-free survival was 84 months. Of 59 patients, 18 (30.5%) died and 41 (69.5%) survived at the time of data analysis.

All samples were immunohistochemically analyzed for STING and cGAS expression (Figure 1 and Figure 2, Table 2).

Elevated STING expression in tumor cells was positively associated with advanced stage and pathologically positive lymph nodes (pN+) in the bivariate analysis (chi-square test, *p* = 0.0139 and 0.0458, respectively) (Table 3).

Additionally, given the small sample size, Fisher’s exact test was used to compare the tumor cell-specific expression of STING against clinically relevant advanced stages of laryngeal cancer, grouping the tumor specimen stage IV against stages I to III and advanced pT3 and pT4 against pT1 and pT2. In addition, intrinsic STING expression in lymph node-positive tumors was compared to that in lymph node-negative tumors. Advanced laryngeal cancer stage IV, pT3, and pT4 cancer specimens, as well as lymph node-positive tumors, were positively associated with elevated intrinsic STING expression in tumor cells (Fisher’s exact test, *p* = 0.0031, 0.0336, and 0.0371, respectively). Finally, dedifferentiated tumors of histological grades G2 and G3 were compared to well-differentiated G1 tumors. Higher histological grade of laryngeal cancer was also positively associated with elevated tumor cell-intrinsic STING expression (Fisher’s exact test, *p* = 0.0204).

After adjusting for age, sex, location, and cGAS expression, increased STING expression was significantly associated with advanced stage IV tumors in a multiple logistic regression model (β = 1.849, SE = ±0.8643, *p* = 0.0324).

As patients with advanced stages of laryngeal cancer have lower overall survival, high STING expression was correlated with lower OS (Fisher’s exact test, *p* = 0.0258).

Tumors that were exclusively localized to the glottis had significantly lower STING expression (Fisher’s exact test, *p* = 0.0337) than supraglottic, subglottic, or transglottic tumors. Of the 11 supraglottic and two subglottic laryngeal cancers, only one tumor sample was stage II, whereas the others were all in advanced stages III and IV. Conversely, 19 of the 46 glottic laryngeal tumor samples were stages I and II, and the remaining were stages III and IV. Significantly more tumors located in the glottis were characterized as stages I and II, pT1 and pT2, pN0, and negative for LVI and PNI (Fisher’s exact test, *p* = 0.0099, 0.0431, 0.0034, 0.0033, and 0.0317, respectively). Thus, there was a moderate negative correlation between stage, pN, and LVI and the glottic location of the tumor (Spearman R = −0.29, −0.35, −0.47, −0.35; *p* = 0.024, 0.007, 0.0003, 0.008, respectively).

Laryngeal tumor specimens were analyzed for cGAS expression. Twenty-two of the 59 specimens had elevated tumor cell-specific staining for cGAS compared to the normal adjacent tissue of the same specimen. However, elevated cGAS expression was not correlated with any clinicopathological characteristics or STING expression (Supplementary Table S1).

**Figure 3:**
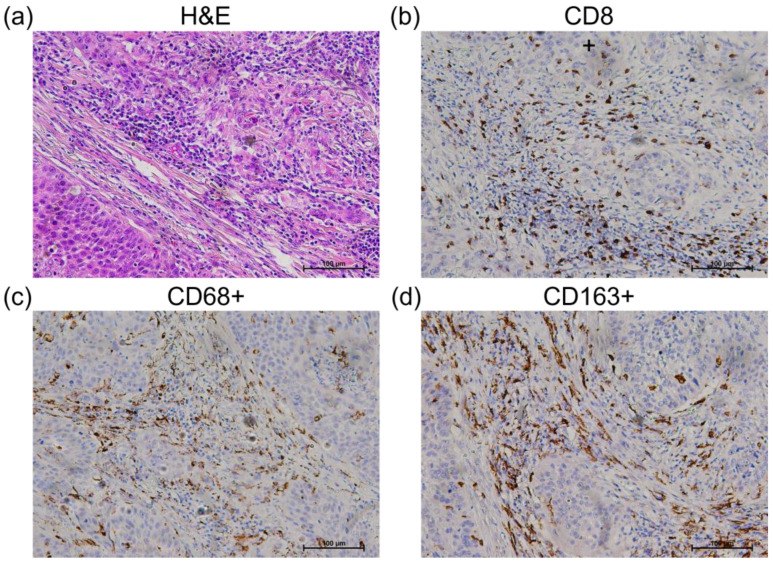
Hematoxylin and eosin staining, CD8, CD68 and CD163 immunostaining. Representative histologic images of immune cell infiltration (H&E) (**a**), CD8+ (**b**), CD68+ (**c**) and CD163+ immune cells (**d**) (200× magnification).

Histological analysis revealed an abundance of immunological cells in the tumor microenvironment that were predominantly positive for STING expression, with more than 50% positive immune cells in all samples, regardless of the STING expression level in tumor cells (Figure 1). The samples were additionally stained for CD8+ T cells and for CD68+ and CD163+ macrophages. In accordance with the histological analysis, all samples had more than 50 positive CD8+, CD68, and CD163+ cells in hotspots per filed at 400× magnification which is considered a high abundance of CD8+ T cells and CD68+ and CD163+ macrophages (Figure 3).

Further quantification of immune cells revealed no significant difference in immune cell composition in the tumor microenvironment of tumors with low or high STING or cGAS expression, respectively (Table 4 and Table S2).

## 4. Discussion

Despite the paradigm shift brought about by the use of immune checkpoint inhibitors (ICIs) in stage IV metastatic head and neck cancers, including laryngeal cancers, the response and OS rates remain modest [25]. Consequently, there is an urgent need for a better understanding of laryngeal cancer at the cellular and molecular levels as well as the complex immune interactions and their changes over time between the tumor itself and the host, both in the immediate tumor environment and at the systemic level. The discovery of novel prognostic and predictive biomarkers is important in the metastatic setting of the disease, as well as in the early stages of laryngeal cancer, considering the psychosocial component of functional loss of the organ. Considering the pronounced heterogeneity of laryngeal tumors, their immunosuppressive microenvironment, and their ability to evade innate and adaptive immune surveillance, it is important to delve into the potentially unique and specific molecular immune mechanisms of laryngeal cancer. The cGAS–STING signaling pathway plays an important role in the activation of the innate immune response but can also influence the differentiation of T lymphocytes, thus affecting the activation of acquired immunity [16]. Extrinsic STING expression by immune cells and intrinsic tumor cell-specific expression of STING play distinct roles in tumor microenvironment modulation, generally inducing antitumor effects through the infiltration of CD8+ T cells as well as the polarization of M2 to M1 macrophages [26,27,28]. Moreover, the STING pathway regulates DNA damage control mechanisms, and hence the response to DNA-damaging therapies [29,30]. A new generation of therapies targeting the cGAS–STING signaling pathway, including STING agonists, have recently attracted interest regarding the antitumor role of cGAS–STING signaling in head and neck carcinomas. The activity of the cGAS–STING axis is important for the effects of standard DNA-damaging therapies such as cisplatin and radiation because the cGAS–STING axis can affect the production of reactive oxygen species (ROS) and the activity of PARP inhibitors [28]. The use of STING agonists as monotherapy and in combination with ICIs and DNA-damaging therapies (radiotherapy and cisplatin) is under investigation in preclinical and clinical studies [27]. A whole-genome CRISPR-Cas9 screen identified STING as a critical intrinsic regulator of the tumor cell response to radiotherapy [28].

Radiotherapy and cisplatin are the most important and commonly used therapeutic choices for unresectable advanced stages of laryngeal cancer and in earlier stages of the disease, especially considering the importance of laryngeal preservation. Despite a relatively good response, some patients develop drug resistance and develop disease progression. Eight patients (14%) were diagnosed with local or systemic recurrence, indicating a significant need for better disease control. Novel therapeutic options, including STING agonists, are potential candidates for improving the treatment outcomes. To the best of our knowledge, no studies have explored the direct protein expression of STING in laryngeal tumors. The goal of this study was to evaluate the expression levels of cGAS and STING in the tumor cells of non-metastatic laryngeal cancer. Previously published TCGA-based studies investigated STING expression in head and neck tumors at the transcriptomic level but did not discriminate between the impact of intrinsic tumor cell-specific STING signaling and cGAS–STING activation in immune cells. These studies included all head and neck tumor sites without discriminating between primary tumor sites [28,31]. In addition, TCGA studies demonstrated that in oropharyngeal squamous cell carcinoma, STING expression decreased with an increase in disease stage, which is in accordance with previous findings in tumors unrelated to the head and neck [18,19,20,21,22]. The results of our study clearly demonstrate a specific increase in intrinsic tumor cell STING expression with the progression of laryngeal cancer. A total of 10 of the 12 patients (83.3%) with stage IV laryngeal cancer had high STING expression levels. In addition, our results demonstrated abundant immune infiltrate in all investigated tumors, which is in accordance with a recent TCGA study that demonstrated a highly dense and fairly consistent proportion of immune cells in samples, regardless of the magnitude of the tumor mutational burden. In TCGA samples, PD L1 expression was significantly higher in high-risk patients, as defined by six genes related to tumor mutational burden [32]. This finding is in line with our results, considering that increased STING expression in the advanced stages of the disease can positively affect PD L1 expression [33]. Given that the overall STING-positive immune infiltrate was abundant in all our samples, transcriptomic analysis would not be suitable for distinguishing between the high level of STING expression in tumor cells and the expression of STING in immune cells present in the tumor microenvironment. Because of the high density of the immune infiltrate in our specimens, specific quantification, that is, a differential distinction or infiltration proportion difference of the immune cells, could not be performed on hematoxylin and eosin-stained slides or for the immunohistochemical staining of CD8+ T cells and CD68+ and CD163+ macrophages, which were all highly represented in cancer samples. This is not surprising in the context of two earlier studies that demonstrated that CD8+ T cells, CD68+, and CD163+ macrophages do not show an infiltration proportion difference in high- and low-risk TCGA cohort patients and do not correlate with high mutational burden, PD L1 expression, or affect the outcomes [32,34]. A differential abundance of distinct immune cells in laryngeal cancer was demonstrated for plasma, T follicular helper, and T regulatory cells in the low-risk group, as well as for M0 macrophages in the high-risk group of TCGA patient cohort [32]. In addition to this, Han et al. compared the influence of immune infiltration on the outcomes of patients with laryngeal cancer. Of the 22 cell types explored, high infiltration of M1 macrophages, dendritic cells (DC), and CD4+ T cells were associated with a statistically significant prolongation of survival in laryngeal cancer [34]. Similar to our findings in laryngeal cancer, STING expression was not correlated with tumor-infiltrating lymphocytes in clear cell renal carcinoma [35]. CSG-3 signature metastatic bladder cancer, characterized by highly expressed genes important for functional cGAS–STING activation, has the highest overall level of tumor-infiltrating leukocytes and is consequently associated with better overall survival with regard to immunotherapy [23]. Across different tumor types in TCGA database, a positive correlation between cGAS and STING RNA expression was found only in CSG-3 tumors with a high cGAS–STING functional activation signature [23]. Although the laryngeal cancer samples in this study demonstrated an inflamed phenotype with abundant intratumoral immune infiltrates, we did not observe a correlation between STING and cGAS expression at the protein level. Therefore, future analyses of downstream responder gene expression are needed to clarify whether laryngeal cancer exhibits functional cGAS–STING activation. One significant role is to determine the radiochemosensitivity of STING-expressing tumors and the efficacy of STING agonists. If STING expression in tumor cells is low, the tumor develops resistance to DNA damage-directed therapy, which is an important concern in laryngeal cancer treatment [28]. In addition, STING expression is important for the effect of PARP inhibitors, which have shown efficacy in clinical studies and are used in clinical practice in patients with BRCA germline mutations, mostly in ovarian, prostate, pancreatic, and breast cancer [27,36,37]. As previously demonstrated, PARP inhibitors can modulate the immune response through the cGAS–STING signaling pathway, even in cells without germline BRCA mutations [38]. The activity of PARP inhibitors in combination with ICIs, chemotherapy, and STING agonists is currently under investigation in preclinical and clinical settings [27,39,40]. Considering that laryngeal carcinomas have a high mutational burden due to frequent mutations in genes responsible for DNA repair, and in line with our results that demonstrate an increase in STING expression in the advanced stages of the disease, it is conceivable that laryngeal cancer is a good candidate for the use of PARP inhibitors and STING agonists in metastatic and non-metastatic advanced stages of the disease and in combination with standard therapy (radiotherapy, chemotherapy, chemoradiotherapy, and ICIs) in different timeline settings (primary, neoadjuvant, and adjuvant treatment). New clinical trials have combined the use of STING agonists with ICIs and RT [27,39,40]. Patients with laryngeal cancer are known to respond well to chemoradiotherapy, which is important not only in stage III but also in other stages of the disease, considering the importance of organ preservation. Our study demonstrates that unlike other tumors of the head and neck, there is an increase in tumor cell-specific STING expression in laryngeal cancer in the advanced stages of the disease, which could be important for the introduction of novel therapeutic options. The limitations of our study, which may affect the interpretation of the results, include its retrospective design and relatively small number of patients. The retrospective design of this study may have led to patient selection and a subsequent bias in the results. Further prospective studies are required to confirm our findings.

## 5. Conclusions

The discovery of new prognostic and predictive biomarkers would help in the development of more precise therapies or combination protocols with increased effectiveness in laryngeal cancer. With respect to previous findings in other tumors and based on the results of this study, high STING expression in laryngeal cancer is a favorable biomarker candidate for potential therapeutic approaches employing STING agonists and ICIs in combination with DNA-damaging therapies (radiotherapy, cisplatin, and PARP inhibitors). STING expression should be considered as a predictive biomarker in future clinical trials.

## Figures and Tables

**Table 1:** Clinicopathological characteristics of patients with LC.

Characteristics		Patients N = 59	%
Age, years (IQR)	65.0 (58.0–71.0)		
	<70	42	71.2
	≥70	17	28.8
Gender	Male	56	94.9
	Female	3	5.1
Location	Supraglottis	11	18.6
	Glottis	46	78.0
	Subglottis	2	3.4
Transglottis	Yes	22	37.3
	No	37	62.7
Stage	I	11	18.6
	II	9	15.3
	III	27	45.8
	IV	12	20.3
pT	T1	11	18.7
	T2	12	20.3
	T3	31	52.5
	T4	5	8.5
pN	Nx, N0	43	72.9
	N1	5	8.5
	N2	10	16.9
	N3	1	1.7
Metastasis	Yes	4	6.8
	No	55	93.2
Histological grade	G1	16	27.1
	G2	37	62.7
	G3	6	10.2
Lymphovascular invasion (LVI)	Yes	15	25.4
	No	44	74.6
Perineural invasion (PNI)	Yes	7	11.9
	No	52	88.1
Overall survival	Yes	41	60.5
	No	18	30.5

**Table 2:** Immunohistochemical analysis for STING and cGAS expression.

Expression		Patients N = 59	%
STING	Low	33	55.9
	High	26	44.1
cGAS	Low	37	62.7
	High	22	37.3

**Table 3:** Clinicopathological characteristics of patients with low and high STING expression.

Characteristics		Low STING N = 33 (%)	High STING N = 26 (%)	*p*-Value
Age	years (IQR)	64.5 (56.5–70.25)	65.0 (58.0–71.5)	
	<70	22 (66.7)	20 (76.9)	0.5636 ^§^
	≥70	11 (33.3)	6 (23.1)	
Gender	Male	31 (93.9)	25 (96.1)	>0.9999 ^§^
	Female	2 (6.1)	1 (3.9)	
Location	Supraglottis	4 (12.1)	7 (26.9)	0.3341 ^‡^
	Glottis	28 (84.9)	18 (69.2)	
	Subglottis	1 (3.0)	1 (3.9)	
Transglottis	Yes	10 (30.3)	12 (46.2)	0.2805 ^§^
	No	23 (69.7)	14 (53.8)	
Stage	I	8 (24.2)	3 (11.5)	0.0139 ^‡^
	II	7 (21.2)	2 (7.7)	
	III	16 (48.5)	11 (42.3)	
	IV	2 (6.1)	10 (38.5)	
pT	T1	8 (24.2)	3 (11.45)	0.0952 ^‡^
	T2	9 (27.3)	3 (11.45)	
	T3	15 (45.5)	16 (61.5)	
	T4	1 (3.0)	4 (15.4)	
pN	Nx, N0	28 (84.8)	15 (57.7)	0.0458 ^‡^
	N1	3 (9.1)	2 (7.7)	
	N2	2 (12.1)	8 (30.7)	
	N3	0 (0.0)	1 (3.9)	
Histological grade	G1	13 (39.4)	3 (11.45)	0.0566 ^‡^
	G2	17 (51.5)	20 (76.9)	
	G3	3 (9.1)	3 (11.45)	
Lymphovascular invasion (LVI)	Yes	5 (15.2)	10 (38.5)	0.0693 ^§^
	No	28 (84.8)	16 (61.5)	
Perineural invasion (PNI)	Yes	5 (15.6)	2 (8.3)	0.4490 ^§^
	No	28 (84.4)	24 (91.7)	
cGAS	Low	21 (63.6)	16 (61.5)	0.4317 ^§^
	High	12 (30.3)	10 (38.5)	
Overall survival	Yes	27 (81.8)	14 (53.8)	0.0258 ^§^
	No	6 (18.2)	12 (46.2)	

^§^ Fisher’s exact test, ^‡^ Chi-square test. The *p*-value represented in bold is statistically significant.

**Table 4:** Immune cell abundance in samples with low and high STING expression.

Characteristics		Low STING N = 33 (%)	High STING N = 26 (%)	*p*-Value
Inflammation	0	0 (0)	0 (0)	0.7778 ^‡^
	+	11 (33.3)	11 (42.3)	
	++	16 (48.3)	11 (42.3)	
	+++	6 (18.2)	4 (15.4)	
CD8+ T cell	0	0 (0)	0 (0)	0.5029 ^‡^
	+	5 (15.2)	5 (19.2)	
	++	19 (57.6)	11 (42.3)	
	+++	9 (27.3)	10 (38.5)	
CD68+ Macrophages	0	0 (0)	0 (0)	0.1154 ^‡^
	+	2 (6.1)	3 (11.5)	
	++	24 (72.7)	12 (46.2)	
	+++	7 (21.2)	11 (42.3)	
CD163+ Macrophages	0	0 (0)	0 (0)	0.3810 ^‡^
	+	4 (12.1)	6 (23.0)	
	++	18 (54.6)	10 (38.5)	
	+++	11 (33.3)	10 (38.5)	

+ low, ++ medium, +++ high number of cells. ‡ Chi-square test.

## Data Availability

Data is contained within the article or supplementary material. Further inquiries can be directed to the corresponding author.

## Supplementary Materials

The following supporting information can be downloaded at: https://www.mdpi.com/article/10.3390/cancers15133510/s1, Table S1. Clinicopathological characteristics of patients with low and high cGAS expression; Table S2. Immune cell abundance in samples with low and high cGAS expression.
