# Supplementary material for: Elevated Tumor Cell-Intrinsic STING Expression in Advanced Laryngeal Cancer
Source: Cancers (Basel). 2023 Jul 5;15(13):3510. doi: 10.3390/cancers15133510 (PMC10341367; doi:10.3390/cancers15133510)
Supplement: Supplementary file 1 [file cancers-15-03510-s001.zip › cancers-2449751-supplementary.pdf]

Supplementary Table S1. Clinicopathological characteristics of patients with low and high cGAS expression.

Characteristics		LOW cGAS N = 37 (%)	HIGH cGAS N = 22 (%)	*p*-value
Age	years (IQR)	66.0 (61.0-73.5)	61.5 (55.0-65.7)	
	< 70	24 (64.9)	18 (81.8)	0.2369§
	≥ 70	13 (35.1)	4 (18.2)	
Gender	Male	35 (94.6)	21 (95.5)	>0,9999§
	Female	2 (5.4)	1 (4.5)	
Location	Supraglottis	8 (21.6)	3 (13.6)	0.7129‡
	Glottis	28 (75.7)	18 (81.8)	
	Subglottis	1 (2.7)	1 (4.6)	
Transglottis	Yes	15 (40.5)	7 (31.8)	0.5845§
	No	22 (59.5)	15 (68.2)	
Stage	I	5 (13.5)	6 (27.3)	0.5020‡
	II	7 (18.9)	2 (9.1)	
	III	17 (46.0)	10 (45.5)	
	IV	8 (21.6)	4 (18.2)	
pT	T1	5 (13.5)	6 (27.3)	0.5251‡
	T2	9 (24.3)	3 (13.6)	
	T3	20 (54.1)	11 (50.0)	
	T4	3 (8.1)	2 (9.1)	
pN	Nx, N0	27 (73.0)	16 (72.8)	0.5882‡
	N1	3 (8.1)	2 (9.1)	
	N2	7 (18.9)	3 (13.6)	
	N3	0 (0.0)	1 (4.5)	
Histological grade	G1	13 (35.1)	3 (13.6)	0.0644‡
	G2	19 (51.4)	18 (81.8)	
	G3	5 (13.5)	1 (4.5)	
Lymphovascular invasion (LVI)	Yes	5 (27.0)	5 (22.7)	0.7673§
	No	32 (73.0)	17 (77.3)	
Perineural invasion (PNI)	Yes	5 (15.6)	2 (9.1)	0.7019§
	No	32 (84.4)	20 (90.9)	
STING	Low	21 (56.8)	12 (54.5)	0.4317§
	High	16 (43.2)	10 (45.5)	
Overall survival	Yes	27 (73.0)	14 (63.6)	0.5614§
	No	10 (27.0)	8 (36.4)	

§ Fisher's exact test, ‡ Chi-square test.

Supplementary Table S2. Immune cell abundance in samples with low and high cGAS expression.

Characteristics		LOW cGAS N = 37 (%)	HIGH cGAS N = 22 (%)	*p*-value
Inflammation	0	0 (0)	0 (0)	0.2819‡
	+	16 (43.3)	6 (27.3)	
	++	14 (37.8)	13 (59.1)	
	+++	7 (18.9)	3 (13.6)	
CD8+ T cell	0	0 (0)	0 (0)	0.4634‡
	+	6 (16.2)	4 (18.2)	
	++	21 (56.8)	9 (40.9)	
	+++	10 (27.0)	9 (40.9)	
CD68+ Macrophages	0	0 (0)	0 (0)	0.1419‡
	+	4 (10.8)	1 (4.5)	
	++	25 (67.6)	11 (50)	
	+++	8 (21.6)	10 (45.5)	
CD163+ Macrophages	0	0 (0)	0 (0)	0.5607‡
	+	7 (18.9)	2 (9.1)	
	++	18 (48.7)	11 (0.5)	
	+++	12 (32.4)	9 (40.9)	

+ low, ++ medium, +++ high number of cells. ‡ Chi-square test
